# A historical note: Rediscovering an unpublished response to Korn and Freidlin (2011)

**DOI:** 10.1177/09622802251403371

**Published:** 2026-01-28

**Authors:** Hongjian Zhu, William F Rosenberger, Feifang Hu, Sofia Villar

**Affiliations:** 1 SVP, Global Head of Statistics and Data Science, SystImmune, Inc., Abb Vie Inc, 4730 Deepwater Ln, SUGARLAND, TX, USA; 2 Department of Statistics, 3298 George Mason University, Fairfax, VA, USA; 3 Department of Statistics, 8367 University of Virginia, Charlottesville, VA, USA; 4 Institute of Public Health, Robinson Way Forvie Site, Cambridge, United Kingdom of Great Britain and Northern Ireland

**Keywords:** Two-armed trial, patient benefit, binary outcome, clinical trial, trial design

## Abstract

Despite extensive research, the use of response-adaptive randomization (RAR) in clinical trials has remained controversial. Korn and Freidlin’s 2011 article reignited this debate back then, prompting numerous responses, including one by Zhu, Rosenberger, and Hu that remained unpublished until now. This article features the original response by Zhu, Rosenberger, and Hu, providing a valuable opportunity to revisit the original arguments, examine subsequent developments in RAR methodology, and offer a more complete historical perspective on this enduring debate. The piece also includes a concluding section by one of the special issue’s co-editor that explores the nuances and complexities of RAR implementation in the context of contemporary clinical trial design.

N.B. Note written and edited by Sofia S. Villar, co-editor of the special issue on the Theory and Practice of Response-Adaptive Randomisation in Clinical Trials: Current and Future Perspectives.

Despite having seen significant research efforts to address concerns regarding the use of response-adaptive randomization in clinical trials as well as sound arguments put forward to move past the debate, the controversy around its use in clinical trials continues to appear insoluble. Korn and Freidlin’s 2011 paper, “*Outcome-adaptive randomization: Is it useful?*,” is an example of an article which reignited this debate 14 years ago, prompting numerous responses. One of these responses, written by Zhu, Rosenberger, and Hu, has remained unpublished until now, due to missing the journal’s four-week deadline for letters to the editor. This special issue will now feature that previously unpublished response, offering a valuable opportunity to revisit the debate, examine subsequent developments, and provide a more accurate historical record of the existing perspectives when the debate first appeared.

The original 2011 piece is presented below. To complement its content—some of which is echoed in other articles within this special issue—a concluding section, “*Is Response-Adaptive Randomisation Useful?*”—The Devil Is in the Details,” written by this co-editor, is appended.

## Outcome-Adaptive Randomization: It Is Useful

To the Editor: In a recent article in *Journal of Clinical Oncology*, Korn and Freidlin^1^ simulated a single outcome-adaptive randomization procedure, drew conclusions about the usefulness of outcome-adaptive randomization in clinical trials, and titled the article accordingly. An accompanying editorial^2^ and additional correspondence^3^ described the limited nature of Korn and Freidlin’s investigation and described both logistical and statistical aspects of outcome-adaptive randomization largely ignored by the paper.

The purpose of this correspondence is to give some guidance from the recent literature on the subject, demonstrate that one can find an outcome-adaptive randomization procedure that does offer improvements over standard designs in certain instances explored by Korn and Freidlin, and describe appropriate simulation techniques that allow for accurate comparison of procedures.

The Korn and Freidlin paper is a simulation study of one procedure, Thall and Wathen’s modification^4^ of a calculation from Thompson’s 1933 paper^5^ that provides the probability that one binomial probability is larger than another when there is a uniform prior distribution. The procedure described by Thall and Wathen^4^ is simply an ad hoc procedure that attempts to place more patients on the better treatment by computing the probabilities by Thompson^5^ and mapping them to a randomization function that reduces the variability of the procedure. Many other outcome-adaptive randomization functions have been described over the past decade in the literature, some based on ad hoc considerations, and others based on formal optimality. Unfortunately, most of these procedures were not referenced or compared by Korn and Freidlin. They compared Thall and Wathen’s procedure only to fixed randomization procedures in 1:1 or 2:1 ratios. They also failed to reference a 2006 book on the subject^6^ that describes many of these functions, compares them by simulation and theory, and makes recommendations.

In the context of Korn and Freidlin’s article, the primary goal of outcome-adaptive randomization is to assign more patients to the better treatment. In the context of a comparison of binomial probabilities between two treatments, one metric of success would be the expected number of treatment failures. On page 122 of Hu and Rosenberger,^6^ some guidelines on when such procedures are appropriate are given. First, the procedure should allow standard inferential tests to be used at the end of the trial. Second, power for the overall treatment comparisons should be preserved. Third, the trial should be fully randomized to prevent bias. They then conclude that, if these three considerations are met, an outcome-adaptive randomization procedure is useful if the expected number of treatment failures is reduced over standard randomized designs. Preserving power and assigning most patients to the better treatment are sometimes competing goals, because wide discrepancy in sample sizes on the two treatment arms can lead to significant power losses. Therefore, one must obtain a suitable “compromise” design.

These guidelines present a template for comparison of outcome-adaptive randomization procedures. The template is as follows: for a fixed sample size n , simulate the randomization procedure 10,000 times, compute the standard inferential test statistic for a treatment comparison, and find the simulated power of the test. Increase or decrease the sample size and repeat the simulation until a benchmark power is obtained. We will use 80%. Repeat the simulation at this sample size and compute the expected number of treatment failures. When multiple procedures are simulated at similar underlying success probabilities (Korn and Freidlin’s $p_{con}$ and $p_{tre}$ ), a valid comparison of expected treatment failures can be made at a fixed power level. The reason that this comparison is better than a comparison at fixed sample sizes is that one procedure may demonstrate significantly fewer expected treatment failures than another, but at a considerable loss of power. Increasing the sample size of that procedure to reach a nominal power will necessarily increase the treatment failures, and any ethical benefit may be lost.

Hu and Zhang^7^ proposed a particular randomization function that targets any allocation ratio, allows standard inferential procedures, preserves or improves power, and can reduce expected treatment failures. The function is described elsewhere,^8^ and simple numerical examples are also given. We can compare its properties in the context of Korn and Freidlin in Table 1. Here, the Hu and Zhang (HZ) procedure is targeting the allocation ratio^9^ explored by Yuan and Yin,^3^ but the tuning parameter of the HZ procedure^7^ is set to 2 to reduce variability.

**Table 1.** A comparison of Hu and Zhang’s procedure (HZ), Thall and Wathen’s procedure (TW), and 1:1 and 2:1 randomization. Simulation based on 10,000 replications in C++.

$p_{con}$	$p_{tre}$	n	Procedure	Proportion to control (S.E.)	Expected number of failures (S.E.)
0.2	0.35	219	1:1	0.501 (0.033)	159 (6.65)
0.2	0.35	240	2:1	0.334 (0.031)	168 (7.08)
0.2	0.35	218	TW	0.317 (0.080)	152 (7.41)
0.2	0.35	210	HZ	0.428 (0.039)	150 (6.42)
0.3	0.6	68	1:1	0.500 (0.060)	37.4 (4.09)
0.3	0.6	76	2:1	0.334 (0.054)	38.0 (4.35)
0.3	0.6	70	TW	0.318 (0.087)	34.6 (4.49)
0.3	0.6	66	HZ	0.411 (0.056)	34.5 (3.83)
0.4	0.65	97	1:1	0.500 (0.050)	46.1 (4.92)
0.4	0.65	111	2:1	0.333 (0.045)	48.1 (5.21)
0.4	0.65	102	TW	0.312 (0.085)	43.7 (5.33)
0.4	0.65	93	HZ	0.436 (0.039)	42.6 (4.61)

Note that for values of $p_{con}=0.2$ and $p_{tre}=0.35$ , the HZ procedure requires nine fewer patients on average than equal allocation, and results in nine fewer treatment failures on average. In order to obtain 80% power, the TW procedure requires eight more patients than HZ and HZ results in two fewer failures. The variability of the HZ procedure is much smaller than that of the TW, as exhibited by the standard errors. For large treatment differences, $p_{con}=0.3$ and $p_{tre}=0.6$ , the benefits of the HZ procedure are dampened. It requires two fewer patients on average than 1:1 allocation, and results in three fewer failures. The expected number of failures is equivalent between HZ and TW, although the HZ procedure requires four fewer patients to reach an average power of 80%. Similar results are seen for $p_{con}=0.4$ and $p_{tre}=0.65$ .

Our conclusion is that in certain cases, the HZ procedure reduces the sample size required to obtain 80% power, and a modest reduction in expected treatment failures is realized. This is consistent with the conclusions drawn in the recent literature exploring different procedures.^6,8^

Our simulation was limited, and no attempt was made to compare to many other procedures in the literature. But our point was to show that broad-based conclusions about outcome-adaptive randomization should not be made without exploring the literature for other procedures and conducting a valid comparison.

Besides these numerical comparisons that show that certain outcome-adaptive randomization procedures can reduce sample size and expected treatment failures, there are many logistical considerations that must be made in implementing outcome-adaptive randomization. These are outlined and described by Berry in his editorial,^2^ in Chapter 12 of Rosenberger and Lachin’s book,^10^ and in Chapter 8 of Hu and Rosenberger’s book.^6^

Here we have focused on outcome-adaptive randomization for comparing two treatments with binary responses. As pointed out by Berry,^2^ outcome-adaptive randomization has a great potential in multi-armed clinical trials. For multi-armed clinical trial with binary responses,^11,12^ the advantages of outcome-adaptive randomization have been studied both theoretically and numerically. For two-armed^13^ or multi-armed^14^ clinical trials with continuous responses, the benefits of outcome-adaptive randomization have also been demonstrated in the literature.

## Is Response-Adaptive Randomization Useful? The Devil Is in the Details

**Zhu et al**

MRC Biostatistics Unit, University of Cambridge, UK

Zhu, Rosenberger, and Hu’s unpublished response highlights a crucial point that was absent in the published in 2011 responses to the Korn and Freidlin paper, and which is often largely overlooked when the debate reappears. The wide variety of response-adaptive procedures, combined with the critical influence of trial phase and context (including the disease prevalence and severity), renders any simplistic generalizations on this topic inaccurate, and detrimental to informed design choices. This very same point was later reiterated by Villar et al.^15^ and expanded in the Roberston et al.^16^ Statistical Science paper. I will build upon the original piece’s arguments to further demonstrate the importance of this argument.

Korn and Freidlin’s stark conclusion—that response-adaptive randomization (RAR) provides only modest or no benefit to trial participants—hinges on several key assumptions whose substantial influence on this verdict are not explicitly acknowledged as critical. Among the numerous assumptions involved in their conclusion, presented in no particular order, are:

- A (quickly observable) binary outcome measure (or primary endpoint).
- A specific treatment effect of interest (or estimand): the simple difference of success rates.
- Specific ranges of parameter values that define the treatment effects for the binary outcome.
- A particular Bayesian RAR design with a preferred and distinct implementation for Phase II (*tuning* but no burn-in) and for Phase III (burn-in and clipping, but no *tuning* ) characteristics.
- The use of a normal approximation to test for the difference in proportions as well as the assumption that it is a valid analytical method across all presented scenarios. Interestingly, the validity of this assumption and the observed type I error rate are not reported in the simulations presented.
- Specific values for type I error rate and power targeted which also differ across phases (e.g. Phase 2 aims at 10% type I error and 80% power while Phase 3 aims for 2.5% and 90%, respectively) while other values are not considered or presented.
- The designs considered do not allow for early stopping due to efficacy or futility.
- The constraint that the control or standard of care cannot outperform the experimental arm.
- The assumption that RAR does not impact patient accrual by assuming no effect on recruitment rates.

Each of the above assumptions, even when considered individually, can significantly alter the conclusion regarding the value of RAR. This sensitivity is perhaps best demonstrated in Figure 3 by Robertson et al.,^16^ recently revisited as Figure 12.1 by Pin et al.^17^. For ease of reference, we reproduce that figure here (Figure 1). The blue dashed curved line in Figure 1 represents the optimal allocation proportion necessary to maximize statistical power for a binary endpoint under a given parameter configuration, plotted as a function of the treatment success rate $p_{tre}$ (taking values within [0,1] ) while holding the control rate $p_{con}$ fixed. For comparison, the flat solid line shows the universally applied 1:1 ratio.

**Figure 1.**
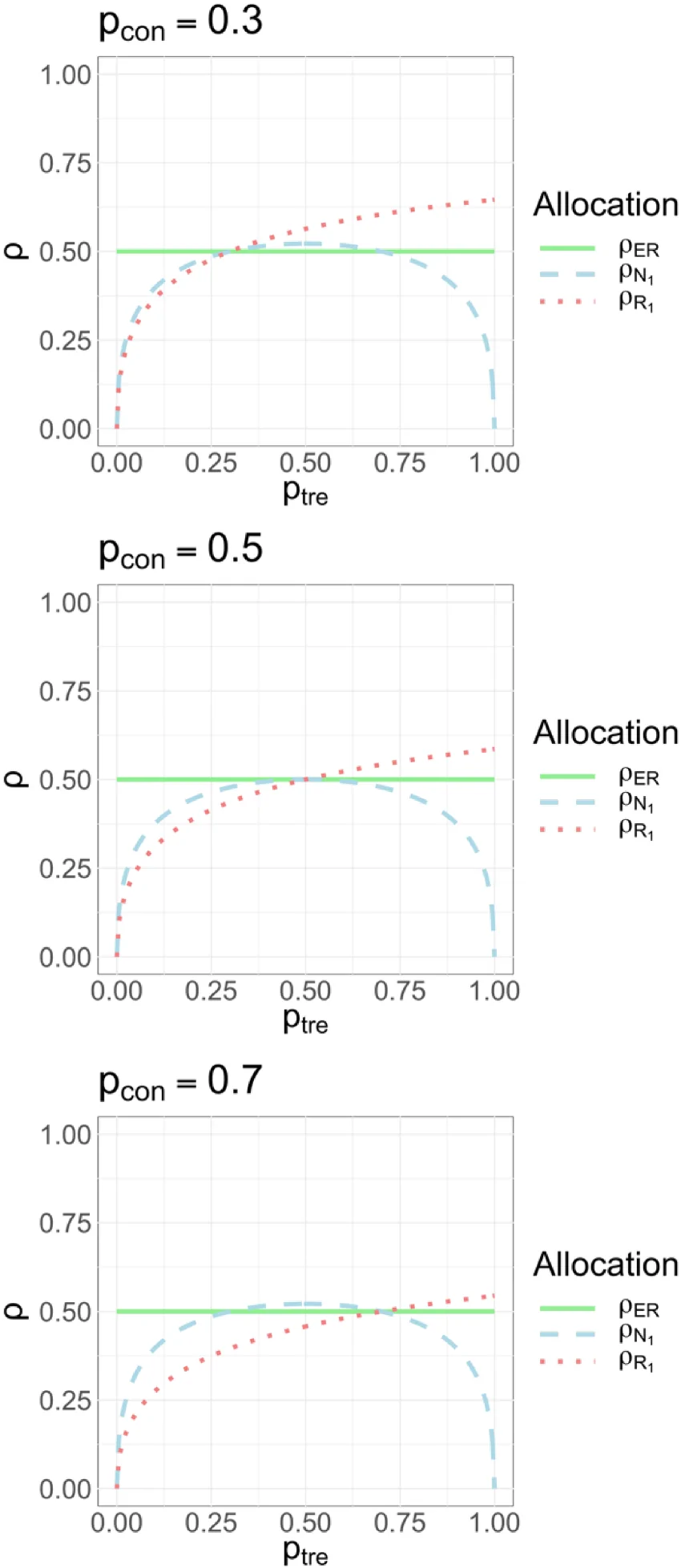
Equal ratio versus optimal allocation ratios as a function of $p_{tre}$ , for $p_{con}\in \{0.3,0.5,0.7\}$ .

The first point that should be noted is that when the primary endpoint is binary, then it is no longer true that: “*1:1 randomization approximately provides the most information about the between-arm treatment effect for a given sample size*.” This will certainly be the case if the two arms have a common and constant variance but this is not true for a binary endpoint particularly when the treatment effect is substantial. Figure 3 by Robertson et al.^16^ clearly shows an equal randomization ratio to be optimal only in those points where the common variance for the test assumption holds. Figure 1) clearly displays the impact of the region in which the treatment effect can lie. The scenarios considered in the Korn and Friedlin (2011) paper are such that $p_{con}<p_{tre}$ and. Interestingly, the scenarios for Phase 2 trials are such that they belong to the region, where the optimal ratio for power and the ethical version by Rosenberger et al.^18^—represented by the red dotted line in Figure 1—move in the same direction (favoring the experimental arm although in different proportions). For the scenarios in Phase 3 trials, the opposite happens, and the effects lie in a region where the optimal ratio for power and the ethical version by Rosenberger et al.^18^ move in the opposite direction (one favoring the control arm and the other the experimental one).

For other endpoints where the common variance assumption is unsuitable (e.g. survival outcomes), the detriment of balancing sample sizes to power remains a similar concern (see Yung et al.^19^ and Pin et al.^20^) for an indepth discussion of optimal ratios for outcome measures beyond binary. Likewise, the choice of a specific estimand (the quantity being estimated) will impact which ratio or region of unbalancing is most helpful for achieving both efficiency and patient benefit. If we were interested in estimating and making inference on the log odds ratio then the optimal ratios for power and ethics are very different from those depicted in Figure 1 and so are these regions that delineate when an unequal ratio may in fact be good for inferential and ethical reasons.

A point that should be noted is that for RAR procedures that are likely to result in considerable deviations from a 1:1 ratio (which were the focus of Korn and Freidlin’s paper) using traditional inference methods can present several challenges. Specifically, for the type of Bayesian RAR considered in that work, it was been shown that one can expect considerable type I error rate inflation with standard approaches (see figures in Appendix 2 of the Villar and Smith paper^21^) and that an inferential approach taking the design into account maybe be more appropriate if type I error rate control is a priority (Baas et al.^22^).

The fact that RAR is likely to work best in combination with early stopping was pointed out by Jennison.^23^ While presenting simulations for RAR procedures used in isolation can be useful to understand this feature, in practice, it is perhaps important to always consider it with the possibility of early stopping. The assumption that the use of RAR procedures does not affect recruitment used in some of the cases presented by Korn and Freidlin appears unlikely in certain settings (see Tehranisa and Meurer^24^) and it critically makes RAR appear less favorable in terms of the time needed to recruit than what it could be. Similarly, the comparison to fixed ratios (as opposed to a RAR procedure) to positively affect recruitment or to result in similar efficient gains at a simpler logistical cost crucially relies on the belief that control cannot be superior to experimental. It is not uncommon to see even in Phase 3 trials the control arm being superior to the experimental one (see e.g. Bousser et al.^25^). If recruitment is positively affected by the use of RAR (or a fixed unequal ratio), the power-benefit tradeoff when comparing this design to competitor designs is also affected and it merits careful consideration in the specific context.

Traditionally, debates on the use of RAR procedures have sought broad conclusions and general recommendations. However, a more fruitful approach may be to examine in depth specific instances, such as two-arm binary endpoint trials. By scrutinizing these simpler cases, we can gain valuable insights into the optimal application of this adaptive tool. In an era where real-time testing of multiple interventions and individualized probability adjustments are increasingly feasible, understanding the historical context of this century-long debate becomes crucial. This co-editor firmly believes that learning from past debate will help unlock the full potential of this and other adaptive designs in modern clinical research.
